# A CT-Based Radiomics Approach to Predict Nivolumab Response in Advanced Non-Small-Cell Lung Cancer

**DOI:** 10.3389/fonc.2021.544339

**Published:** 2021-02-24

**Authors:** Chang Liu, Jing Gong, Hui Yu, Quan Liu, Shengping Wang, Jialei Wang

**Affiliations:** ^1^ Department of Medical Oncology, Fudan University Shanghai Cancer Center, Shanghai, China; ^2^ Department of Oncology, Shanghai Medical College, Fudan University, Shanghai, China; ^3^ Institute of Thoracic Oncology, Fudan University Shanghai Cancer Center, Shanghai, China; ^4^ Department of Radiology, Fudan University Shanghai Cancer Center, Shanghai, China

**Keywords:** CT-based radiomics approach, nivolumab, NSCLC, machine-learning, immunotherapy

## Abstract

**Purpose:**

This study aims to develop a CT-based radiomics model to predict clinical outcomes of advanced non-small-cell lung cancer (NSCLC) patients treated with nivolumab.

**Methods:**

Forty-six stage IIIB/IV NSCLC patients without EGFR mutation or ALK rearrangement who received nivolumab were enrolled. After segmenting primary tumors depicting on the pre-anti-PD1 treatment CT images, 1,106 radiomics features were computed and extracted to decode the imaging phenotypes of these tumors. A L1-based feature selection method was applied to remove the redundant features and build an optimal feature pool. To predict the risk of progression-free survival (PFS) and overall survival (OS), the selected image features were used to train and test three machine-learning classifiers namely, support vector machine classifier, logistic regression classifier, and Gaussian Naïve Bayes classifier. Finally, the overall patients were stratified into high and low risk subgroups by using prediction scores obtained from three classifiers, and Kaplan–Meier survival analysis was conduct to evaluate the prognostic values of these patients.

**Results:**

To predict the risk of PFS and OS, the average area under a receiver operating characteristic curve (AUC) value of three classifiers were 0.73 ± 0.07 and 0.61 ± 0.08, respectively; the corresponding average Harrell’s concordance indexes for three classifiers were 0.92 and 0.79. The average hazard ratios (HR) of three models for predicting PFS and OS were 6.22 and 3.54, which suggested the significant difference of the two subgroup’s PFS and OS (p<0.05).

**Conclusion:**

The pre-treatment CT-based radiomics model provided a promising way to predict clinical outcomes for advanced NSCLC patients treated with nivolumab.

## Introduction

Over the past decade, immune checkpoint inhibitors targeting programmed cell death protein-1 (PD-1) or programmed cell death protein ligand-1 (PD-L1) have opened a new epoch of treatment for advanced non-small-cell lung cancer (NSCLC), with improved survival and durable responses compared with chemotherapy in patients both in first- and second-line treatment (1–5). PD-1 or PD-L1 inhibitors pembrolizumab, nivolumab, and atezolizumab, prolonged overall survival (OS) compared with chemotherapy in patients with previously treated advanced NSCLC based on the results of Keynote-010 (1), CheckMate 017/057 (2, 3) and OAK studies (4). The phase III study CheckMate 078 has demonstrated consistent results of superior OS by nivolumab compared with docetaxel in a predominantly Chinese population with previously treated advanced NSCLC (6).

Despite remarkable success of immunotherapy, up to 60% of patients with advanced NSCLC could not benefit from PD-1 or PD-L1 inhibitors (7). Different biomarkers have been investigated to predict the efficacy and prognosis, such as PD-L1 expression and copy number gains (1–4, 8–11), tumor mutation burden (TMB) (12–14), microsatellite instability (MSI) (15), tumor infiltrating lymphocytes (16–18) and inflammatory cytokines (19). Even though the PD-L1 expression of tumor cell has been identified as a predictive biomarker for response of immunotherapy in both newly diagnosed or previously treated NSCLC (3, 4, 11), the relationship between the PD-L1 expression and the therapeutic effects of nivolumab is still unclear. Tumor heterogeneity, instability of tissue specimens, non-standardized detection techniques and the dynamic nature of the immune microenvironment are also limitations of PD-L1 expression as a predictive biomarker (20, 21). The urgent need to discover and validate non-invasive, stable predictive biomarkers to select patients who will benefit from immunotherapy remains an ongoing challenge.

Since non-invasive diagnostic images can depict the phenotypes of lung tumor, recently studies have illustrated that utilization of imaging biomarker to predict the survival stratification of advanced NSCLC patients with different therapies is feasible. Among these non-invasive imaging based prediction or classification models, CT image based radiomics approach has been developed and applied to build the prognostic prediction model for evaluating the effectiveness and necessity of developing different therapies, e.g., targeted therapeutics, chemotherapy, radiation therapy, and for early prediction of clinical outcome. The non-invasive quantitative imaging technique may provide a new approach to assess the clinical outcome at an early stage of updated PD-1 therapeutic process.

**Figure 1:**
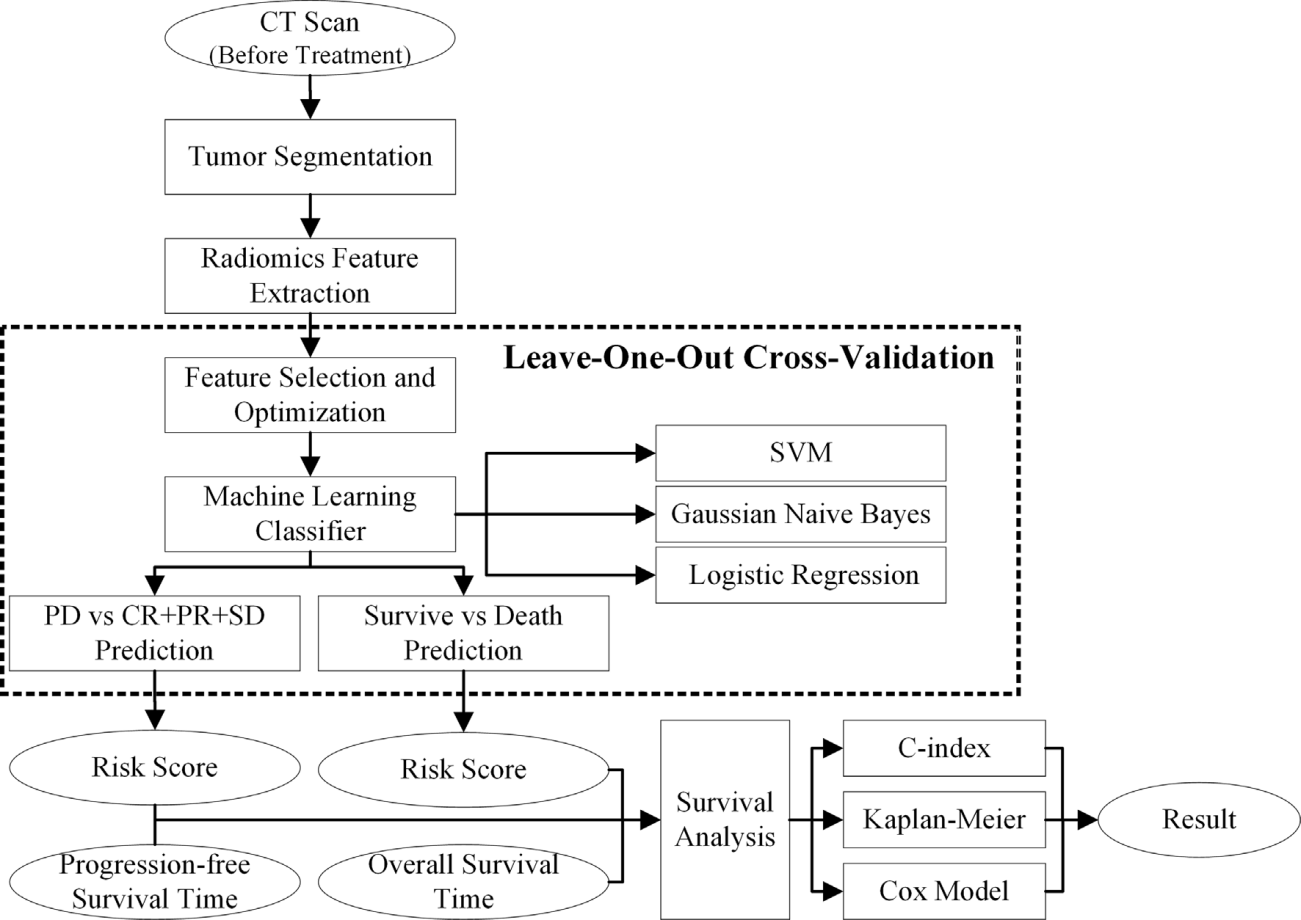
Flowchart of our proposed model.

In this study, we proposed a novel CT-based radiomics model to predict the progression probability to the recommended nivolumab therapy for individually patient. To decode the imaging phenotypes of lung tumor, we computed and extracted thousands of pretherapy CT features to deeply interpret the patients treated with immunotherapy to select critical PD-1/PD-L1 associated phenotypic features. Then, we used three machine-learning classifiers to develop the CT-based radiomics models to stratify the risk of progression-free survival (PFS) and overall survival (OS) in advanced stage NSCLC patients. Finally, we analyzed and compared the Kaplan–Meier survival estimators of the stratified subgroups with high and low risk for progression and death (**Figure 1**).

## Materials and Methods

### Patients

Forty-six patients with previously treated NSCLC were prescribed with nivolumab from CheckMate 078 study, CheckMate 870 study or clinical practice between Apr 2016 and Jan 2019 at Fudan University Shanghai Cancer Center. All patients were histologically or cytologically-diagnosed with locally advanced or metastatic NSCLC. Patients were included regardless of tumor PD-L1 expression. Patients with epidermal growth factor receptor (EGFR)-mutation or anaplastic lymphoma kinase (ALK) translocation-positive tumors were excluded. We retrospectively collected clinical data and treatment outcomes from the patients’ medical records. The clinical stage was assigned according to the 8th edition of the TNM staging system.

The institutional review board of Fudan University Shanghai Cancer Center approved this study.

### Treatment

Patients received intravenous nivolumab at dose of 3 mg/kg or fixed dose of 240mg every two weeks until disease progression or discontinuation owing to intolerance of toxicity. All patients received a diagnostic contrast-enhanced chest CT prior to immunotherapy. All the CT scans were reconstructed by using the standard convolution kernel. The pixel spacing of CT image ranges from 0.672 mm to 0.822 mm, and the slice thickness is 1 mm or 1.5 mm. Each axial slice image was reconstructed with a matrix 512×512 pixels. The pre-treatment CT scan was collected and used as baseline imaging data.

### Efficacy

Efficacy was assessed by determining PFS, OS, overall response rate (ORR) and the disease control rate (DCR). PFS was defined as the time from initiation of nivolumab therapy to disease progression or death. Patients alive without progression at the time of analysis were censored at their last follow-up. OS was defined as the time from initiation of nivolumab therapy to death. DCR was defined as the percentage of patients with a complete response (CR), partial response (PR), and stable disease (SD), while ORR was defined as the percentage with CRs and PRs. The tumor response was initially assessed after 8 weeks of nivolumab therapy and subsequently thereafter every 8 weeks using the Response Evaluation Criteria In Solid Tumors (RECIST, version 1.1). Responses were defined as the best response from the start of treatment until disease progression.

### Statistical Analysis

**Figure 2:**
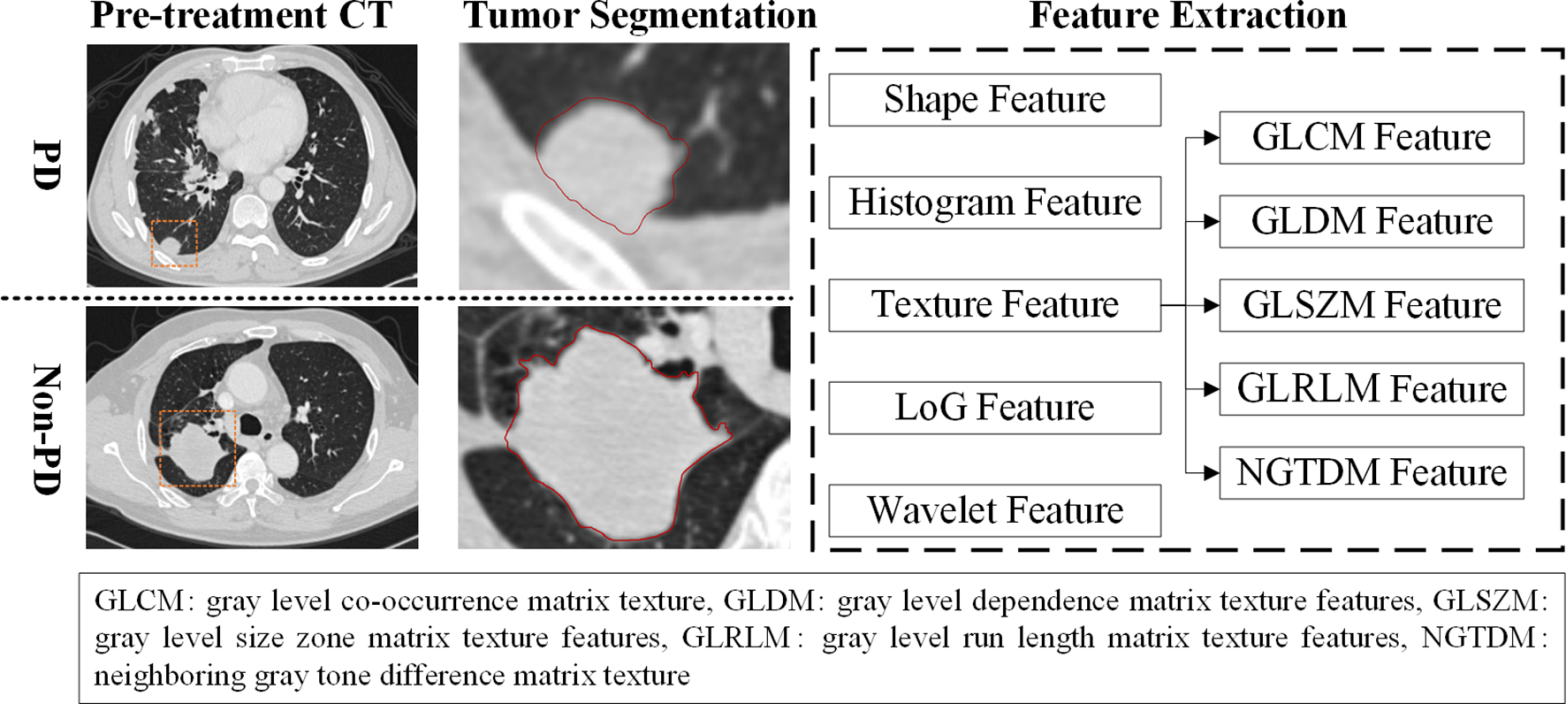
The radiomics feature extraction process.

CT images were first interpreted qualitatively and quantitatively by two radiologists (Dr. Shengping Wang with 15 years experience and Dr. Quan Liu with 28 years experience). **Figure 2** shows the radiomics feature extraction process. To evaluate the therapeutic effect, radiologists provided a standardized report to record lymph node status and common sites of distant metastasis (i.e., bones, liver, and brain) for each patient during the treatment cycles. Then, the radiologists delineated the 3D boundary of each primary tumor and segmented the tumor volume by using the CT scan examined before immune treatment. The largest tumor was defined as target lesion for subject with multiple lesions in this study. All the primary tumors were segmented manually in slice by slice fashion on CT images. Due to the variant of CT parameters, a B-spline curve interpolation algorithm was used to resample the 3D CT images to a spacing of (1, 1, and 1mm). In order to character the imaging phenotypes of each tumor, 1,106 CT based radiomics features were initially computed and extracted to quantitative the tumor. Among these features, 274 LoG features, 728 wavelet features, 14 shape features, 18 histogram features, and 68 texture features were involved. The LoG features were calculated based on image filtered with Laplacian of Gaussian (LOG) filter, and wavelet features were extracted by using image filtered with wavelet filter. Since each phenotypic feature has different value range, a feature normalization technique was used to transform these radiomics features to [0, 1].

Due to a large number of redundant features in the initial feature pool, a L1-based feature selection method was applied to the redundant features and reduce the dimensionality of radiomics features remove. During this process, a linear support vector classifier was used to build the meta-transformer to select the robust imaging features. After feature selection, classification models were built by training three different machine-learning classifiers namely, support vector machine (SVM) classifier, logistic regression classifier (LRC), and Gaussian Naïve Bayes (GNB) classifier, respectively. To evaluate the performance of our proposed models, a leave-one-out cross-validation (LOOCV) was used to train and test the classifier. In order to avoid biases in portioning dataset, the feature selection process and machine-learning classifier were embedded into the LOOCV training/testing cycles.

Finally, several statistical data analysis methods were applied to measure the association between the model’s predicted low or high risk scores and patients’ PFS and OS, which include 1) a Harrell’s concordance index (C-index) analysis, 2) Kaplan–Meier plots, and 3) Cox proportional hazards regression models. To assess the model’s performance, the cases were divided into two groups of low and high risk in cancer progression by applying an operation threshold of 0.5 to the prediction scores generated by three classifiers namely, SVM, NBC, and LRC.

In this study, the prediction models were built by using Python programming software (version 3.6, https://www.python.org), and the statistical data analysis process was implemented on R software (version 3.5.2, https://www.r-project.org). To evaluate the performance of our proposed model, a maximum likelihood based receiver-operating characteristic (ROC) fitting program (ROCKIT, http://www-radiology.uchicago.edu/krl, University of Chicago) was used to compute the area under a ROC curve (AUC) value and the corresponding 95% confidence interval (CI). PFS and OS were estimated by the Kaplan-Meier method, along with hazard ratios (HRs). All outcome measures were calculated with 95% CIs, which were estimated by use of the Cox proportional hazard model. The significance level of statistical tests was set at p < 0.05. All expressed p values and CIs were two-tailed. All the medical image processes and performance evaluation processes were performed on a computer with Intel Core i7-8700 CPU 3.2GHz × 2, 16 GB RAM.

## Results

### Patient Characteristics

A total of 46 patients with previously treated advanced NSCLC were administrated with nivolumab at Fudan University Shanghai Cancer Center between Apr 2016 and Jan 2019. Their baseline characteristics at the initiation of nivolumab therapy are shown in **Table 1**. The patients’ median age was 62.0 years (range, 46 to 77 years). There was a higher proportion of males (34/46, 73.9%) than females, and of current/former smokers (30/46, 65.2%) than never smokers. Thirty-four patients (73.9%) were diagnosed with adenocarcinoma while 12 patients (26.1%) were diagnosed with squamous cell carcinoma; 42 (91.3%) had stage IV disease at baseline. All 46 patients had an Eastern Cooperative Oncology Group performance status (ECOG PS) of 1.

**Table 1:** Baseline patient characteristics (N = 46).

Characteristic	All, No. of patients (%)
Age, median (range), years	62 (46–77)
Sex	
Male	34 (73.9)
Female	12 (26.1)
ECOG PS	
1	46 (100)
Smoking status	
Current/former smoker	30 (65.2)
Never smoker	16 (34.8)
Number of lines of prior systemic cancer therapy	
1	42 (91.3)
≥2	4 (8.7)
Tumor histology	
Adenocarcinoma	34 (73.9)
Squamous cell carcinoma	12 (26.1)
Tumor Stage	
IIIB	4 (8.7)
IV	42 (91.3)
No. of metastatic sites at baseline	
1	22 (47.8)
2	16 (34.8)
≥3	8 (17.4)

ECOG PS, Eastern Cooperative Oncology Group performance status.

All patients had a routine examination before initiation of nivolumab treatment, 22 patients (47.8%) had one metastatic site, 16 patients (34.8%) had two metastatic sites and 8 patients (17.4%) had more than two metastatic sites. In 42 patients (91.3%), nivolumab was used as second-line treatment and in 4 patients (8.7%) as third-line or later treatment.

### Efficacy

Tumor responses are shown in **Table 2**. One patient (2.2%) achieved CR, 6 patients (13.0%) achieved PR and 12 (26.1%) had SD, resulting in an ORR of 15.2% (95% CI, 4.7–25.7%) and a DCR of 43.5% (95% CI, 29.0–58.0%).

**Table 2:** Tumor responses.

Responses	All patients (*n* = 46) (*n* or %)
CR	1 (2.2)
PR	6 (13.0)
SD	12 (26.1)
PD	27 (58.7)
ORR	15.2% (95 CI, 4.7–25.7%)
DCR	43.5% (95% CI, 29.0–58.0%)

CR, complete response; DCR, disease control rate; ORR, objective response rate; PD, progressive disease; PR, partial response; SD stable disease.

**Figure 3:**
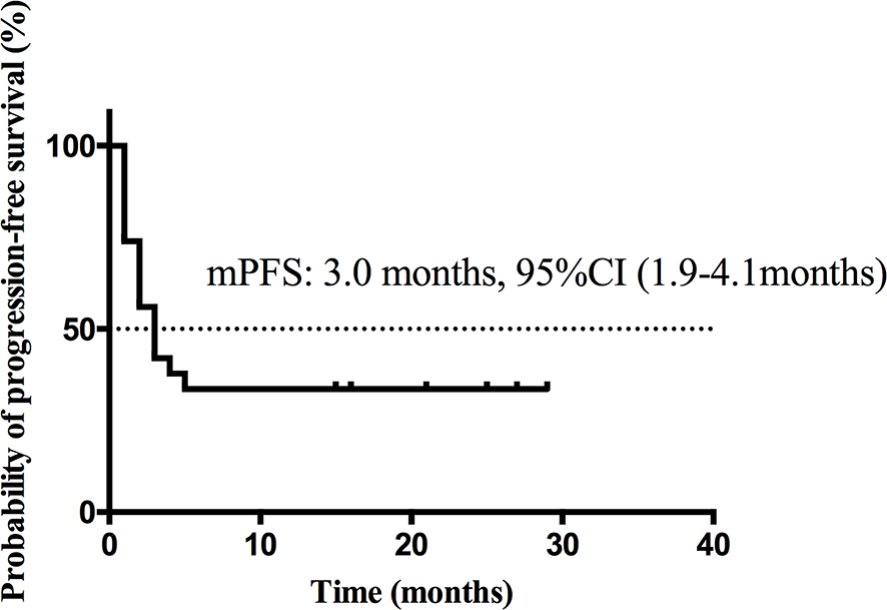
Kaplan-Meier curve of PFS of all patients.

**Figure 4:**
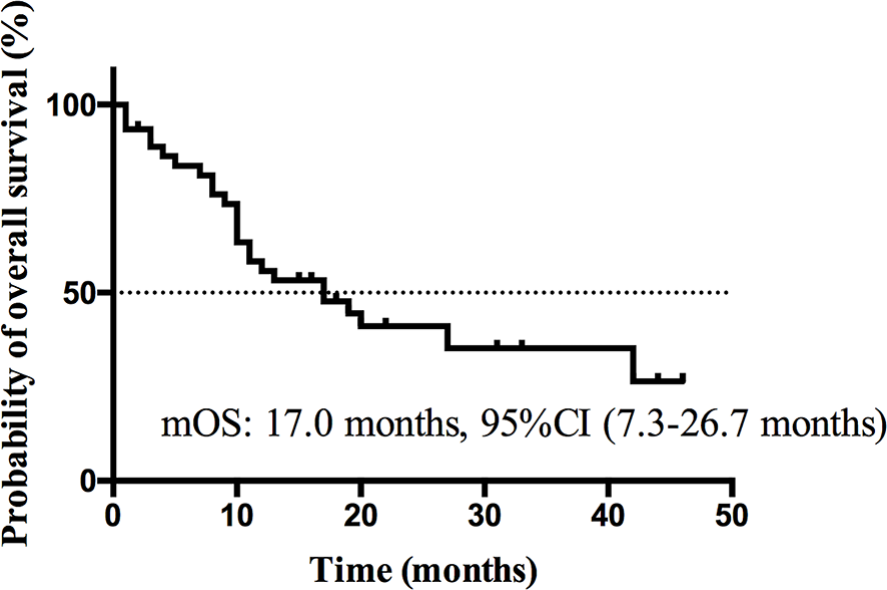
Kaplan-Meier curve of OS of all patients.

At the cutoff date Dec 13^th^ 2019, median follow-up time was 11.5 months (range, 1.0–46.0 months). Nineteen patients (41.3%) were still alive, 2 patients (4.3%) were lost to follow-up and 25 patients (54.3%) were dead at the cutoff date. The median PFS (**Figure 3**) was 3.0 months (95% CI, 1.9 to 4.1 months) and the estimated median OS (**Figure 4**) was 17.0 months (95%CI, 7.3–26.7 months).

### Development of Prediction Model and Survival Analysis

**Figure 5:**
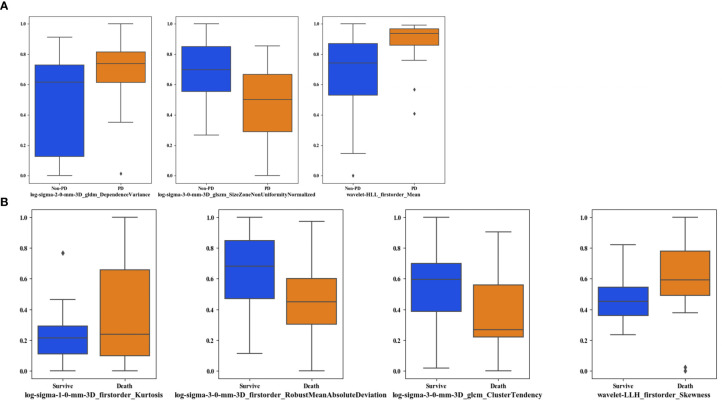
Boxplots of the frequently selected imaging features in the LOOCV process. **(A)** shows the imaging features selected in PFS prediction process, **(B)** shows the imaging features in OS prediction process.

**Figure 5** shows the boxplots of three imaging features frequently selected in LOOCV process. By using the feature selection method, three imaging features were selected from the initial 1,106 feature pool in PFS prediction process. Two LoG image features and one wavelet feature were involved. The boxplots showed that PD and non-PD category have different distributions in three features. It indicated that the selected features had a potential to classify between PD and non-PD cases. Meanwhile, four imaging features were selected to build OS prediction model, involving three LoG image features and one wavelet feature.

**Figure 6:**
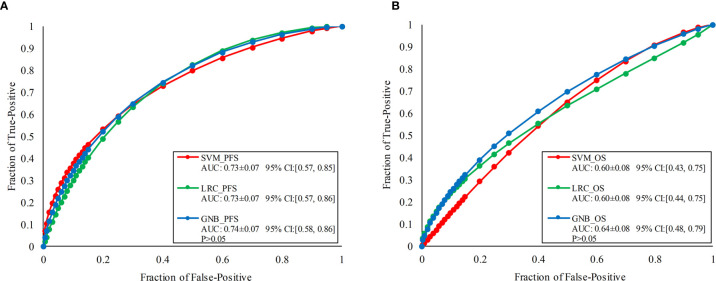
ROC comparisons of PFS and OS classification models built with three classifiers namely, SVM, LRC, and GNB, respectively. **(A)** Illustrates the ROCs of PFS classification models, **(B)** illustrates the ROCs of OS prediction models.

**Figure 6** illustrates the ROC curves of PFS and OS classification models built with three classifiers. To predict the risk of PD, SVM, LRC and GNB generated AUC values of 0.73 ± 0.07 [95% CI: (0.57, 0.85)], 0.73 ± 0.07 [95% CI: (0.57, 0.86)], and 0.74 ± 0.07 [95% CI: (0.58, 0.86)], respectively. Meanwhile, SVM, LRC and GNB generated AUC values of 0.60 ± 0.08 [95% CI: (0.43, 0.75)], 0.60 ± 0.08 [95% CI: (0.44, 0.75)], and 0.64 ± 0.08 [95% CI: (0.48, 0.79)]. To evaluate the inter classifier differences; the p-values of the prediction scores generated by three classifiers were computed by using a univariate z-score test. It showed that the AUC values of three classifiers were no significant difference (p>0.05).

**Figure 7:**
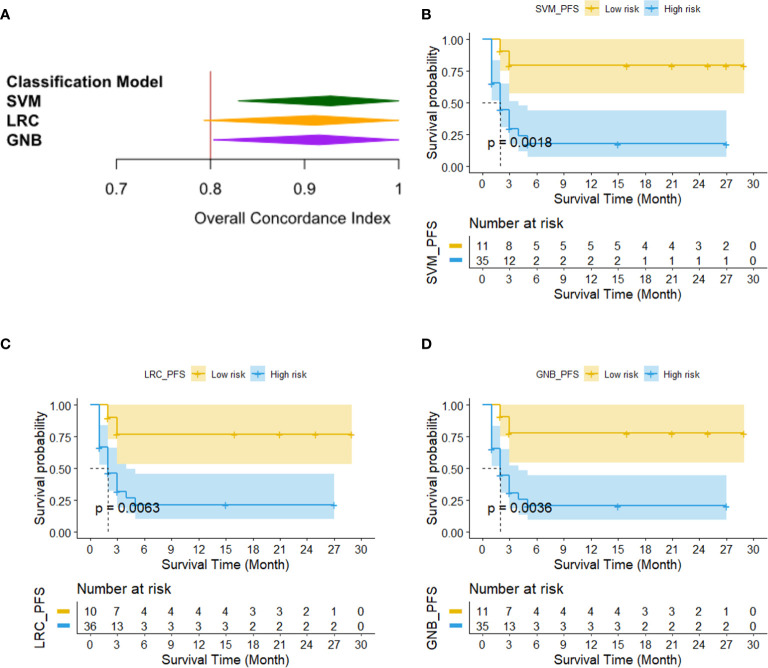
The results of survival analysis of three PFS classification models. **(A)** C-indexes generated by three PFS prediction models, **(B–D)** Kaplan–Meier PFS estimates from all 46 patients by using SVM, LRC and GNB classifier, respectively.

**Figure 7** shows the survival analysis results of three PFS classification models. **Figure 6A** compares the Harrell’s C-indexes for PFS generated by three classifiers. For SVM, LRC and GNB classifier, the C-index was 0.93 [95% CI: (0.83, 1.0)], 0.91 [95% CI: (0.79, 1.0)], and 0.92 [95% CI: (0.80, 1.0)], respectively. **Figures 6B–D** illustrates the Kaplan–Meier plots of PFS by using SVM, LRC and GNB classifier, respectively. The Kaplan–Meier survival curves demonstrated that the low risk cohort predicted by three classifiers was significantly different from the high-risk group by using immune therapy (p<0.05). **Table 3** lists the summary of data analyses of three cox regression models for PFS. The hazard ratios (HR) of three models reach over 5.6, which suggested the dramatic difference of the two subgroup’s PFS in immune treatment (p<0.05).

**Table 3:** Summary of data analyses of three cox regression models for PFS and OS.

	PFS			OS		
	HR	95% CI	p-value	HR	95% CI	p-value
SVM	6.85	(1.61, 29.15)	0.0092	2.95	(1.17, 7.40)	0.021
LRC	5.63	(1.33, 23.85)	0.019	5.17	(2.04, 13.10)	0.00054
GNB	6.18	(1.46, 26.18)	0.013	2.50	(1.03, 6.03)	0.042

**Figure 8:**
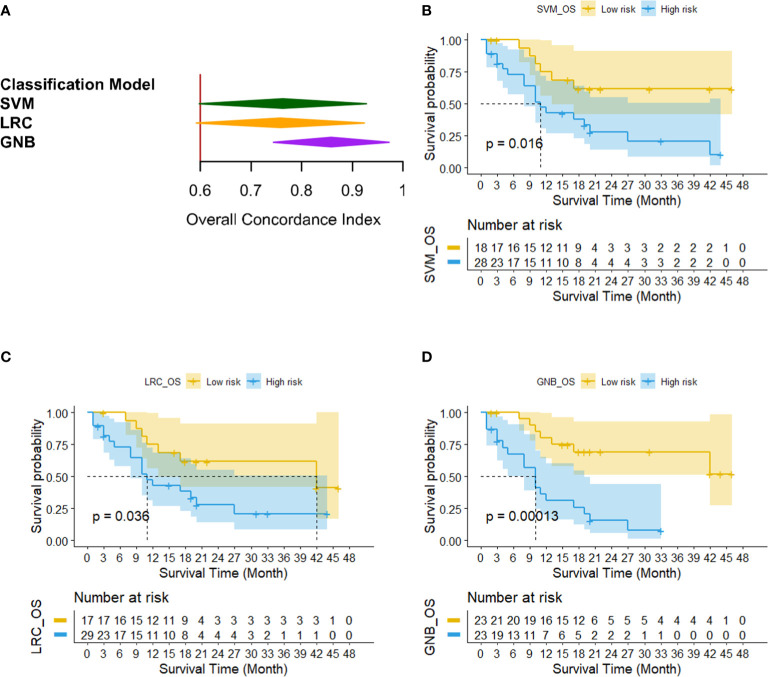
The results of survival analysis of three OS classification models. **(A)** C-indexes generated by three OS prediction models, **(B–D)** Kaplan–Meier OS estimates from all 46 patients by using SVM, LRC and GNB classifier, respectively.

**Figure 8** shows the survival analysis results of three OS classification models. **Figure 8A** compares the Harrell’s C-indexes for OS generated by three classifiers. For SVM, LRC and GNB classifier, the C-index was 0.76 [95% CI: (0.60, 0.93)], 0.76 [95% CI: (0.60, 0.92)], and 0.86 [95% CI: (0.74, 0.97)], respectively. **Figures 8B–D** illustrates the Kaplan–Meier plots of OS by three prediction models. It shows that the low risk OS cohort was significantly different from the high-risk OS group in Kaplan–Meier curve (p<0.05). **Table 3** lists the summary of data analyses of three cox regression models for OS. The hazard ratios (HR) of three models reach over 2.5 (p<0.05).

## Discussion

Although immunotherapy has been a pivotal development in the management of advanced NSCLC, durable responses and improved survival have been observed only in 20–50% of patients (1–5, 11, 22). Predictive biomarkers of response for immunotherapy and superior survival are urgently needed to improve patient selection and avoid toxicity in potential non-responders.

PD-L1 expression is the only biomarker currently approved by the US Food and Drug Administration (FDA) to select patients who are most likely to benefit from immunotherapy. Compared with docetaxel, nivolumab demonstrated better overall survival, with PD-L1 expression conferring enhanced efficacy in pretreated patients with advanced non-squamous NSCLC in Checkmate 057 study (3). However, among patients with advanced, previously treated squamous-cell NSCLC in Checkmate 017 study (2), OS, ORR, and PFS were significantly better with nivolumab than with docetaxel, regardless of PD-L1 expression level. The survival benefit with nivolumab was also observed regardless of PD-L1 expression level in Chinese patients with previously treated NSCLC in Checkmate 078 study (6). Another study explained that PD-L1 expression alone was insufficient to determine whether patients should receive PD-1 or PD-L1 blockade therapy (23). Furthermore, more and more studies demonstrated that there were many factors associated with the PD-L1 expression, including copy number gains (24), heterogeneity (25), dynamic changes (20) and other participants of immune cell subsets (26–28) in NSCLC. In addition to PD-L1 expression, recent research indicated that TMB of 10 or more mut/Mb was associated with improved response and prolonged PFS in both tumor PD-L1 expression 1% or greater and less than 1% subgroups and was thus identified as a potential biomarker for first-line therapy of nivolumab plus ipilimumab in advanced NSCLC (29). Although research on predictors of response to immunotherapy has sprung up, there are still few well-recognized non-invasive biomarkers of immunotherapy with high-specificity, high-sensitivity and stability.

To assess the immunotherapy response, irRECIST (Immune-related Response Evaluation Criteria in Solid Tumors), iRECIST and imRECIST (Immune-Modified Response Evaluation Criteria In Solid Tumors) were proposed (30–33). In the previously reported studies, PET/CT based response evaluation models have been investigated and developed to evaluate the short-term or long-term response of immunotherapy for lung cancer (34, 35). These studies evaluated the treatment response of immunotherapy effectively, but series PET/CT images during the immunotherapy process were needed to analyze to build prediction models. In recent years, numerous studies have evaluated the potential clinical utility of radiomics features from CT images of NSCLC, correlated with tumor histology, staging and patient prognosis (36–48). Nardone V et al. (37) used pre- and post-contrast CT sequences to contour the gross tumor volume (GTV) of the target lesions prior to nivolumab treatment. The impact of variations on contouring was analyzed by two delineations, which were performed on each patient, and the CT texture analysis (TA) parameters were tested for reliability using the Intraclass Coefficient Correlation method (ICC). The study indicated that TA parameters could identify patients that will benefit from PD‐1 blockage by defining the radiological settings that were potentially suggestive of an active immune response. Xu et al. (41) evaluated deep-learning networks for predicting clinical outcomes through analyzing time-series CT-images of locally advanced NSCLC patients. In our study, only pre-immunotherapy CT images were used to evaluate and predict the response results of immunotherapy. Thus, the treatment response might be predicted before conducting the immunotherapy by using CT images.

In this study, a non-invasive CT-based radiomics model was developed to predict the effectiveness of immunotherapy for advanced NSCLC patients. Thousands of quantitative imaging features were computed and investigated to decode the phenotypes of primary lung tumor. Then, the optimal feature pool selected from initial radiomics features were used to train and test three machine-learning classifiers to build prognosis prediction models. A LOOCV method was applied to test and evaluate the model performance. The results demonstrated that it was an effective way to predict the effectiveness of immunotherapy for advanced NSCLC patients by using machine-learning based models (i.e., results showed in **Figure 6**). If our models were robust by testing on the more diverse and larger dataset in future studies, it would provide a new way to predict patient’s short-term treatment response before immunotherapy prescribed in the advanced lung cancer.

To further investigate that how much extra benefit we could obtain for predicting individual patient’s PFS and OS by using the risk scores predicted by our machine-learning models, we also analyzed the survival analysis to evaluate and compare the outcomes of patients with different risk factor. Three machine-learning classifiers yielded high concordance with clinical evaluation outcomes determined by independent radiology review (IRR) for predicting PFS (i.e., C-index for SVM: 0.93, LRC: 0.91, and GNB: 0.92) and OS (i.e., C-index for SVM: 0.60, LRC: 0.60, and GNB: 0.64). Then, the Kaplan–Meier survival analysis illustrated significant difference between the high and low risk patient group for PFS and OS analysis (p<0.05).

Despite of promising results, our study had some limitations. Firstly, to develop CT radiomics models, three machine-learning classifiers were trained and tested on a relatively small dataset with only forty-six cases. Although the LOOCV method was applied in the classifier training and testing process to avoid biases, the robustness and effectiveness of our model were still needed to be evaluated by using more diverse and larger data sets. Secondly, only CT-based radiomics features were used to predict the PFS and OS of advanced NSCLC patients. Some of the other potentially useful clinical information and image features (i.e., biomarkers, MRI image, PET image) have not been explored. Thus, different kinds of features needed to be investigated in our future studies. Thirdly, only the selected target lesions were analyzed instead of all the lesions; nevertheless, the degree of enhancement after CT enhanced scanning of target lesions in different tissues will be different. Lastly, this was only a primary technology development study that just developed a CT-based radiomics model to predict clinical outcomes of advanced NSCLC patients treated with nivolumab. Due to the incomplete data of retrospective studies, we did not include clinical data, genomics and other factors for analysis. Before our prediction models were applied into clinical practice, we will conduct more clinical validation studies to improve the performance of prediction model by combining imaging technologies, clinical characteristics, genomics and other factors.

In conclusion, the novel CT-based radiomics model has the ability to predict the progression probability for patients with advanced NSCLC receiving nivolumab therapy.

## Data Availability Statement

The raw data supporting the conclusions of this article will be made available by the authors, without undue reservation.

## Ethics Statement

The studies involving human participants were reviewed and approved by the institutional review board of Fudan University Shanghai Cancer Center. Written informed consent to participate in this study was provided by the patient/participants.

## Author Contributions

JW and SW designed the study, collected data and verified its integrity, and helped writing the manuscript. CL, JG, and HY were responsible for statistical analysis and writing the manuscript. SW and QL were responsible for interpreting CT images and verifying the integrity of the data. All authors contributed to the article and approved the submitted version.

## Funding

This study was sponsored by the Natural Science Foundation of Shanghai (No. 19ZR1410400), China Postdoctoral Science Foundation (No. 2019M651372), and National Natural Science Foundation of China (No. 82001903).

## Conflict of Interest

The authors declare that the research was conducted in the absence of any commercial or financial relationships that could be construed as a potential conflict of interest.
